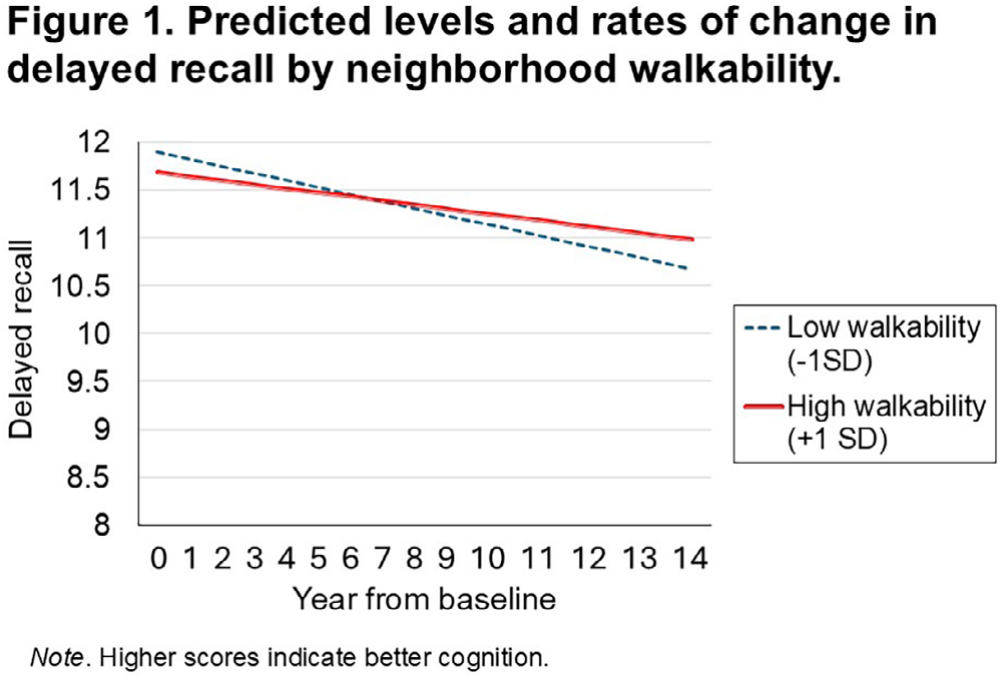# Neighborhood walkability, healthy food environments, and cognitive decline over 14 years among midlife women: Study of Women's Health Across the Nation

**DOI:** 10.1002/alz70860_097856

**Published:** 2025-12-23

**Authors:** Jinshil Hyun, Bradley M. Appelhans, Charles B Hall, Emma Barinas‐Mitchell, Rebecca C. Thurston, Carrie A. Karvonen‐Gutierrez, Monique Hedderson, Imke Janssen, Carol A. Derby

**Affiliations:** ^1^ Albert Einstein College of Medicine, Bronx, NY, USA; ^2^ Rush University, Chicago, IL, USA; ^3^ University of Pittsburgh, Pittsburgh, PA, USA; ^4^ University of Pittsburgh School of Medicine, Pittsburgh, PA, USA; ^5^ University of Michigan, Ann Arbor, MI, USA; ^6^ Kaiser Permanente, Oakland, CA, USA

## Abstract

**Background:**

Neighborhood physical environments may shape health behaviors, ultimately impacting cognitive health outcomes. However, previous studies have yielded mixed results, and few studies have included long‐term follow‐ups. The present study aims to investigate whether neighborhood walkability and healthy food environments are associated with longitudinal cognitive decline among women over midlife.

**Method:**

We examined effects of walkability and healthy food environments on rates of decline in processing speed (Digit Symbol Substitution Test), working memory (Digit Span backwards), and immediate and delayed episodic memory (East Boston Memory Test) among 1391 women (49% non‐Hispanic White, 24% Black, 12% Chinese, 15% Japanese) enrolled in the Study of Women's Health Across the Nation (SWAN). Women were 49‐60 years old at cognitive baseline, and mean follow‐up was 10.2±3.5 years. Neighborhood walkability was assessed using the EPA's National Walkability Index. Healthy food environments were assessed using the CDC's Modified Retail Food Environment Index (mRFEI). Covariates were baseline age, race/ethnicity, education, study site, and time‐varying financial strain, smoking, alcohol use, hormone use, and menopause status.

**Result:**

Women living in areas with higher walkability showed slower rates of decline in immediate (estimate=0.019, SE=0.009) and delayed recall (estimate=0.018, SE=0.008) (Figure 1). The pattern of results stayed similar after controlling baseline physical activity and cardiovascular risk factors. A significant walkability × financial strain interaction (estimate=0.194, SE=0.077) suggested that women exhibited lower immediate recall when they experienced higher financial strain, but only in areas with low walkability. For healthy food environments, there were no significant associations with any cognitive domains. However, significant three‐way food environments × race × time interactions for working memory (estimate=0.031, SE=0.013) and delayed recall (estimate=0.056, SE=0.022) suggested that healthy food environments were protective against more accelerated cognitive decline among Japanese women compared to White women.

**Conclusion:**

Midlife women living in more walkable areas experienced slower decline in episodic memory over the 14‐year period spanning midlife. Healthy food environments showed different effects on cognitive decline across racial/ethnic groups. Future studies should investigate precise mechanisms that underline the observed associations to identify targeted policy recommendations and intervention strategies.